# Supplementary material for: Chemical Composition of Ducrosia flabellifolia L. Methanolic Extract and Volatile Oil: ADME Properties, In Vitro and In Silico Screening of Antimicrobial, Antioxidant and Anticancer Activities
Source: Metabolites. 2022 Dec 31;13(1):64. doi: 10.3390/metabo13010064 (PMC9866066; doi:10.3390/metabo13010064)
Supplement: Supplementary file 1 [file metabolites-13-00064-s001.zip › metabolites-2098080-supplementary.pdf]

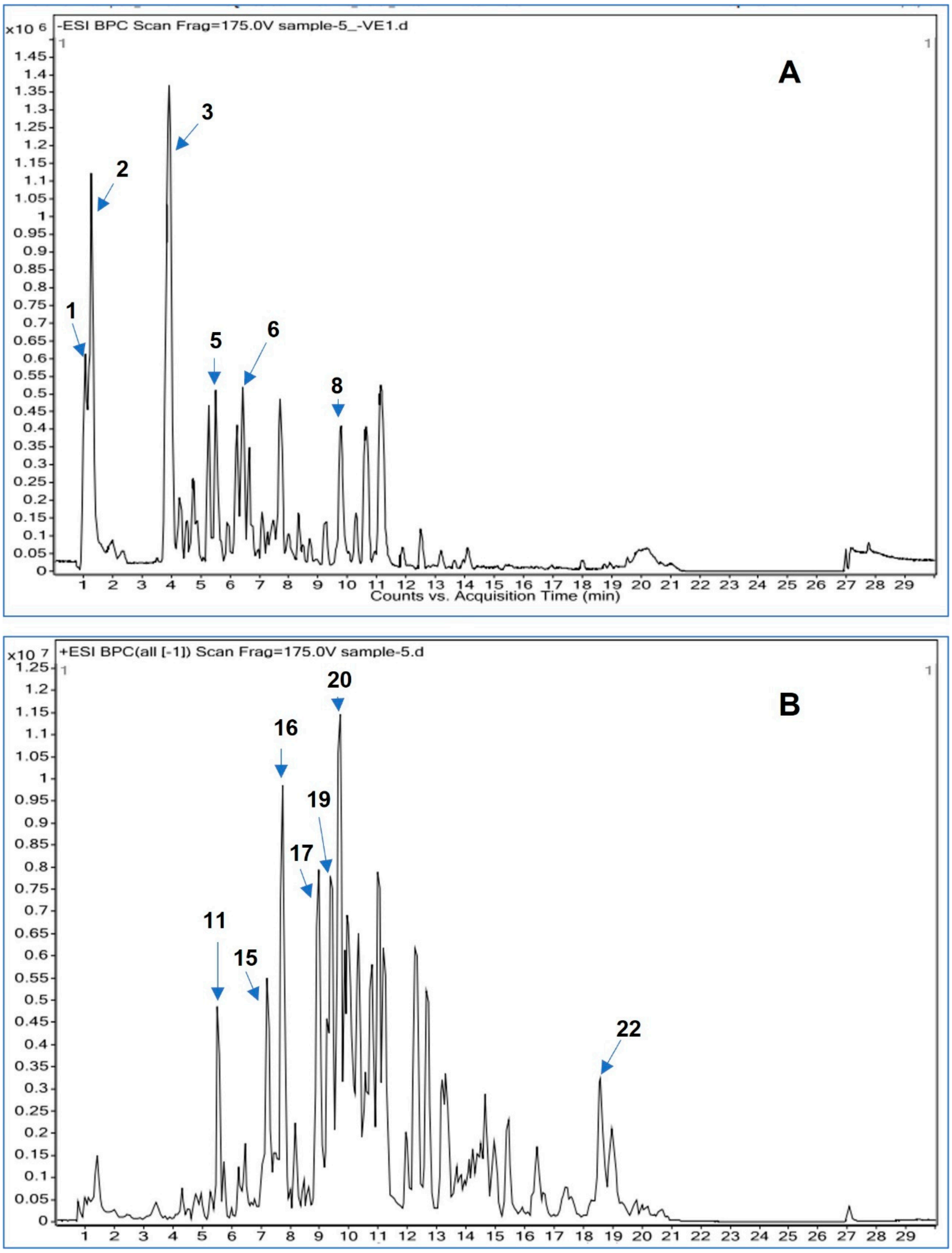

Supplementary material Figure S1. HR-LCMS chromatograms (A: positive chromatobram, B: negative chromatogram) showing the main compounds identified in *D. flabellifolia* methanolic extract. Numbers on the chromatograms correspond to the compounds listed in Table 2.

Supplementary material Table S1. Tripeptides identified by HR-LCMS technique in *D. flabellifolia* methanolic extract.

N°	Compound Name	RT (mn)	MW (g/mol)	Chemical Formula	[*m*/*z*]-	[*m*/*z*]+
**1**	Asn Asn Asn	0.961	360.1396	$C_{12} H_{20} N_6 O_7$	341.1214	-
**2**	Thr Gln Glu	1.094	376.1512	$C_{14} H_{24} N_4 O_8$	411.1207	-
**3**	His Asn Cys	4.233	372.1184	$C_{13} H_{20} N_6 O_5 S$	353.1003	-
**4**	Phe Glu Gln	4.93	422.1812	$C_{19} H_{26} N_4 O_7$	439.1393	-
**5**	Gln Pro His	5.402	380.1818	$C_{16} H_{24} N_6 O_5$	361.1637	-
**6**	Thr Thr Trp	5.533	406.1855	$C_{19} H_{26} N_4 O_6$	423.1443	-
**7**	Gln Met Val	5.959	376.1777	$C_{15} H_{28} N_4 O_5 S$	411.1465	-
**8**	Ser Asn Leu	5.967	332.171	$C_{13} H_{24} N_4 O_6$	367.1404	-
**9**	Arg Gly Gly	6.213	288.1552	$C_{10} H_{20} N_6 O_4$	323.1244	-
**10**	Ile Trp Asp	6.768	432.2005	$C_{21} H_{28} N_4 O_6$	467.1697	-
**11**	Ser Ser Glu	7.214	321.12	$C_{11} H_{19} N_3 O_8$	-	322.1272
**12**	Trp Ile Asp	7.382	432.2014	$C_{21} H_{28} N_4 O_6$	467.1706	-
**13**	Asp Leu Trp	7.646	432.2024	$C_{21} H_{28} N_4 O_6$	467.1717	-
**14**	Thr Glu Ser	8.895	335.1357	$C_{12} H_{21} N_3 O_8$	-	336.1429
**15**	Asp His Phe	9.207	417.1652	$C_{19} H_{23} N_5 O_6$	-	422.1438
**16**	Thr Asp Ser	10.346	321.1206	$C_{11} H_{19} N_3 O_8$	-	304.1172
**17**	Val Asp Cys	10.553	335.1153	$C_{12} H_{21} N_3 O_6 S$	-	340.0942

RT: Retention Time (mn); MW: Molecular Weight (g/mol); [m/z]-: mass-to-charge ratio in negative ionization mode; [m/z]+: mass-to-charge ratio in positive ionization mode.

Supplementary material Table S2. Binding affinity, conventional hydrogen-bonding, the number of closest interacting residues and distance to closest interacting residue (Å) of the compound with best scores with the different targeted receptors (1HD2, 2XCT, 2QZW, AND 3LN1).

**1HD2**					
**Compounds**	**Binding Affinity ($kcal \times mol^{-1}$)**	**Conventional H-Bonds**	**No. Closest Interacting Residues**	**Closest Interacting Residue**	
				**Residue**	**Distance (Å)**
Asn Asn Asn	−5.8	9	7	Arg86	1.908
Thr Gln Glu	−5.7	9	6	Glu16	2.061
His Asn Cys	−5.7	8	7	Glu16	2.355
Phe Glu Gln	−6.4	6	6	Val94	2.017
Gln Pro His	−6.4	6	5	Glu16	2.073
Thr Thr Trp	−6.2	7	6	Glu16	2.251
Gln Met Val	−5.3	7	6	Glu16	1.938
Ser Asn Leu	−5.4	7	7	Glu16	2.116
Arg Gly Gly	−5.5	7	6	Glu16	2.075
Ile Trp Asp	−6.8	7	5	Arg86	2.107
Trp Ile Asp	−6.7	8	7	Arg86	1.977
Asp Leu Trp	−6.4	7	4	Arg86	1.997

Ser Ser Glu	−4.4	8	7	Val70	1.951
Thr Glu Ser	−4.5	9	4	Glu16	1.818
Asp His Phe	−4.6	6	5	Gly92	2.032
Thr Asp Ser	−4.3	7	7	Val67	2.163
Val Asp Cys	−4.1	5	6	Lys63	2.209
10-Hydroxyloganin	−5.9	7	7	Val94	2.177
2,4,6,8,10-dodecapentaenal	−4.5	4	6	Asn122	1.925
2-Phenylaminoadenosine	−6.7	4	7	Arg86	1.822
Atractyloside	−6.8	8	8	Gly92	2.153
Cortisol 21-sulfate	−6.6	4	5	Val75	1.760
5,8,11-heptadecatriynoic acid	−5.3	2	5	Val94	1.989
Galactan	−5.8	8	7	Glu16	2.131
Harderoporphyrin	−6.4	5	3	Pro100	2.047
Ergoline-1,8-dimethanol, 10-methoxy-6-methyl-, (8b)-	−3.7	2	5	Glu16	2.250
Ecgonine-methyl ester	−5.3	3	3	Lys63	2.052
2-Hydroxy-3-(4-methoxyethylphenoxy)- propanoic acid	−4.9	5	6	Val70	2.141
Lomatin	−4.0	2	5	Lys63	2.254
Marmesin	−4.5	2	4	Asn21	2.197
Purpurogallin	−5.2	5	3	Val70	1.948
Atranorin	−4.4	3	4	Lys63	1.937
Methyl 7- desoxypurpurogallin-7-carboxylate trimethyl ether	−4.3	3	5	Lys63	2.226
Dihydro-Obliquin	−4.4	2	4	Asn24	1.840
13-amino-tridecanoic acid	−3.6	3	4	Asn21	2.028
Gummiferol	−4.2	4	3	Lys63	2.863
Farnesyl pyrophosphate	−4.8	4	6	Gly92	1.960
Syringic acid	−4.6	3	6	Val70	3.538
Khayanthone	11.3	0	1	Val70	1.476
**2XCT**					
**Compound**	**Binding Affinity (kcal×mol^{-1})**	**Conventional H-Bonds**	**No. Closest Interacting Residues**	**Closest Interacting Residue**	
				**Residue**	**Distance (Å)**
Asn Asn Asn	−6.0	7	5	Arg517	1.958
Thr Gln Glu	−6.0	4	5	Asp1024	2.106
His Asn Cys	−6.0	10	8	Ala540	2.103
Phe Glu Gln	−6.6	9	8	Ser1021	1.975
Gln Pro His	−6.4	7	7	Asp1024	2.058
Thr Thr Trp	−6.7	6	5	Glu465	2.080
Gln Met Val	−5.9	7	8	Ser1021	1.899
Ser Asn Leu	−6.5	5	7	Glu1017	2.094
Arg Gly Gly	−5.7	11	8	Asp510	2.229
Ile Trp Asp	−6.7	6	10	Arg517	2.186
Trp Ile Asp	−6.9	5	6	Gln541	2.187
Asp Leu Trp	−6.5	7	8	Asp1024	1.962
Ser Ser Glu	−5.0	8	5	Ala1094	1.893

Thr Glu Ser	−5.6	6	6	His1081	1.924
Asp His Phe	−6.8	6	5	Ser1021	2.430
Thr Asp Ser	−6.1	6	6	Asp1024	2.132
Val Asp Cys	−5.5	8	7	Arg629	2.478
10-Hydroxyloganin	−7.6	6	4	Asp1105	1.904
2,4,6,8,10-dodecapentaenal	−4.7	2	5	Ser1028	2.640
2-Phenylaminoadenosine	−7.3	5	7	Gln541	2.093
Atractyloside	−7.1	5	5	His1046	2.112
Cortisol 21-sulfate	−6.8	6	5	Arg1047	1.981
5,8,11-heptadecatriynoic acid	−4.9	2	5	Asp1037	2.616
Galactan	−7.1	7	7	Arg1033	1.936
Harderoporphyrin	−8.2	6	5	Arg1377	2.186
Ergoline-1,8-dimethanol, 10-methoxy-6-methyl-, (8b)-	−6.9	3	6	Arg1485	2.446
Ecgonine-methyl ester	−5.1	4	5	Lys466	2.302
2-Hydroxy-3-(4-methoxyethylphenoxy)- propanoic acid	−5.7	3	5	Lys1043	2.046
Lomatin	−7.2	2	3	Gln541	2.043
Marmesin	−7.1	3	6	Lys1043	2.134
Purpurogallin	−6.6	5	4	Ser1021	2.220
Atranorin	−6.9	7	7	Ser1173	1.811
Methyl 7- desoxypurpurogallin-7-carboxylate trimethyl ether	−6.5	4	5	Arg1309	2.073
Dihydro-Obliquin	−7.1	3	5	Arg629	2.034
13-amino-tridecanoic acid	−4.5	3	4	Gln1095	1.978
Gummiferol	−5.6	3	6	Asn1269	2.082
Farnesyl pyrophosphate	−5.7	4	6	Asp1096	1.978
Syringic acid	−5.3	4	8	Arg629	2.193
Khayanthone	−7.2	2	4	Arg1048	2.232
**2QZW**					
**Compound**	**Binding Affinity (kcal×mol-1)**	**Conventional H-Bonds**	**No. Closest Interacting Residues**	**Closest Interacting Residue**	
				**Residue**	**Distance (Å)**
Asn Asn Asn	−6.5	8	6	Asp86	2.029
Thr Gln Glu	−6.9	10	11	Gly34	2.011
His Asn Cys	−7.1	10	10	Gly85	1.818
Phe Glu Gln	−7.9	8	12	Asn131	1.935
Gln Pro His	−7.7	7	7	Arg195	2.291
Thr Thr Trp	−7.7	8	8	Asp86	2.060
Gln Met Val	−6.4	5	7	Thr221	2.207
Ser Asn Leu	−7.0	9	7	Thr222	1.964
Arg Gly Gly	−6.3	10	7	Gly220	2.111
Ile Trp Asp	−8.4	7	8	Asp218	2.039
Trp Ile Asp	−7.3	7	10	Asp86	1.860
Asp Leu Trp	−7.4	8	9	Arg192	2.174
Ser Ser Glu	−6.4	8	5	Thr221	1.868
Thr Glu Ser	−6.4	10	11	Asp218	2.257

Asp His Phe	−8.1	9	9	Thr222	2.201
Thr Asp Ser	−5.8	6	5	Asp86	2.056
Val Asp Cys	−6.2	8	5	Asn131	2.172
10-Hydroxyloganin	−7.2	6	7	Asp32	1.997
2,4,6,8,10-dodecapentaenal	−5.5	2	6	Ser88	2.294
2-Phenylaminoadenosine	−8.3	6	9	Arg195	2.223
Atractyloside	−8.3	6	7	Glu132	2.023
Cortisol 21-sulfate	−7.7	5	5	Arg192	1.917
5,8,11-heptadecatriynoic acid	−5.8	3	8	Asn131	2.030
Galactan	−7.2	7	6	Asp32	1.845
Harderoporphyrin	−8.2	6	7	Asp36	1.804
Ergoline-1,8-dimethanol, 10-methoxy-6-methyl-, (8b)-	−6.4	4	6	Ser326	2.126
Ecgonine-methyl ester	−5.1	4	3	Asp138	2.147
2-Hydroxy-3-(4-methoxyethylphenoxy)- propanoic acid	−5.5	6	5	Tyr158	1.905
Lomatin	−7.1	4	5	Lys145	1.965
Marmesin	−6.8	4	3	Arg192	2.577
Purpurogallin	−6.9	6	5	Gln147	2.258
Atranorin	−7.9	6	7	Thr222	1.978
Methyl 7- desoxypurpurogallin-7-carboxylate trimethyl ether	−6.5	3	7	Gly246	1.995
Dihydro-Obliquin	−7.0	2	7	Ser88	2.229
13-amino-tridecanoic acid	−4.4	4	9	Thr222	2.190
Gummiferol	−5.6	3	5	Lys96	2.087
Farnesyl pyrophosphate	−6.7	4	7	Gly34	1.908
Syringic acid	−5.3	5	6	Asn146	1.893
Khayanthone	−7.0	5	7	Asp214	1.894
**3LN1**					
**Compound**	**Binding Affinity (kcal×mol^{-1})**	**Conventional H-Bonds**	**No. Closest Interacting Residues**	**Closest Interacting Residue**	
				**Residue**	**Distance (Å)**
Asn Asn Asn	−7.5	12	10	Gly121	1.907
Thr Gln Glu	−7.7	7	7	Ser34	1.884
His Asn Cys	−5.8	10	9	Gly121	2.350
Phe Glu Gln	−7.9	10	11	Thr198	2.277
Gln Pro His	−8.1	8	7	Asp111	2.047
Thr Thr Trp	−8.5	8	7	Ser34	1.880
Gln Met Val	−7.5	8	6	Gln447	1.993
Ser Asn Leu	−7.4	8	9	Phe196	1.864
Arg Gly Gly	−6.8	10	7	Ala142	1.980
Ile Trp Asp	−8.5	7	4	Ser457	1.963
Trp Ile Asp	−8.3	8	11	Arg29	2.082
Asp Leu Trp	−7.0	8	9	Ser34	2.096
Ser Ser Glu	−6.8	8	8	Ala142	2.105

Thr Glu Ser	−6.1	7	6	Glu222	2.318
Asp His Phe	−8.9	9	13	Ala137	1.909
Thr Asp Ser	−6.7	8	9	Ser34	2.252
Val Asp Cys	−6.4	6	5	Gln275	1.835
10-Hydroxyloganin	−7.2	6	7	Cys32	1.944
2,4,6,8,10-dodecapentaenal	−5.5	2	6	Arg29	2.068
2-Phenylaminoadenosine	−7.9	5	4	Asp333	2.397
Atractyloside	−8.2	4	11	Asn368	2.062
Cortisol 21-sulfate	−8.0	5	7	Gly211	2.315
5,8,11-heptadecatriynoic acid	−6.0	5	12	Asn368	1.936
Galactan	−6.6	10	6	Ser112	2.062
Harderoporphyrin	−9.9	6	7	Ser160	1.898
Ergoline-1,8-dimethanol, 10-methoxy-6-methyl-, (8b)-	−7.2	4	7	Asp43	2.508
Ecgonine-methyl ester	−5.8	5	5	Gln415	2.136
2-Hydroxy-3-(4-methoxyethylphenoxy)- propanoic acid	−6.2	4	2	Val247	1.878
Lomatin	−7.1	3	5	Asn567	2.225
Marmesin	−8.5	3	7	Asn19	2.234
Purpurogallin	−7.5	5	4	Thr198	1.771
Atranorin	−7.1	5	5	Ser457	2.047
Methyl 7- desoxypurpurogallin-7-carboxylate trimethyl ether	−6.9	4	2	His337	2.103
Dihydro-Obliquin	−9.2	1	9	Ser516	2.497
13-amino-tridecanoic acid	−5.5	4	7	Glu451	2.202
Gummiferol	−6.1	5	8	Cys26	1.869
Farnesyl pyrophosphate	−6.8	5	6	Arg319	2.117
Syringic acid	−6.1	4	9	Ala142	2.270
Khayanthone	−7.2	4	5	Asn90	2.080
